# Co-infection of Hepatitis B and Hepatitis C among HIV-infected patients: A cross-sectional study from tertiary care hospital of eastern Nepal

**DOI:** 10.1371/journal.pone.0264791

**Published:** 2022-03-03

**Authors:** Lok Bahadur Shrestha, Gopal K. Yadav, Saugat Pradhan, Abhilasha Sharma, Tejendra Pandit, Roshan Chhetry, Basudha Khanal

**Affiliations:** 1 Department of Microbiology & Infectious Diseases, B. P. Koirala Institute of Health Sciences, Dharan, Nepal; 2 Department of Emergency Medicine, Kalaiya Hospital, Bara, Nepal; 3 Department of Internal Medicine, B. P. Koirala Institute of Health Sciences, Dharan, Nepal; University of Cincinnati College of Medicine, UNITED STATES

## Abstract

**Introduction:**

This study was conducted with an objective to analyze prevalence and risk factors associated with co-infection of hepatitis B virus (HBV) and hepatitis C virus (HCV) in HIV-positive patients with reference to their CD4+ T cell status.

**Materials and methods:**

HIV-positive patients visiting the HIV clinic for CD4+ T cells testing at B.P. Koirala Institute of Health Sciences were tested for Hepatitis B and Hepatitis C. Data regarding age, gender, mode of HIV transmission, duration of HIV diagnosis, antiretroviral therapy status, antiretroviral therapy duration, hepatitis B or C status, and CD4+ T cells count were collected via face-to-face interview, and hospital records. The data were entered in Microsoft Excel 2019 v16.0 (Microsoft, WA, USA) and statistical analysis was performed by using statistical package for social sciences, IBM SPSS® v21 (IBM, Armonk, New York).

**Results:**

Out of 474 HIV-positive patients, HIV-HBV, HIV-HCV, and HIV-HBV-HCV co-infections were seen in 2.95% (14/474), 18.14% (86/474), and 2.53% (12/474) respectively. The primary route of infection was intra-venous drug use (IVDU) in those co-infected with HBV only (8, 57.14%), HCV only (46, 53.49%), and both HBV and HCV (8, 66.67%). HIV patients infected via IVDU were 2.40 times more likely to have HIV-HCV co-infection as compared to those infected via sexual route (AOR 2.40, 95% CI: 1.49,3.86). Similarly, HIV patients with CD4+ T cells count less than 350 cells/mm^3^ were more likely to have HIV-HBV-HCV co-infection as compared to those with CD4 count equal to and more than 350 cells/mm^3^ (AOR 13.84, 95% CI: 2.90,66.10).

**Conclusion:**

HIV-positive patients are at high risk of hepatitis B and/or hepatitis C co-infection. Intravenous drug use, and lower CD4+T cells count are the most important risk predictors of co-infection. All HIV-positive patients should be carefully screened with hepatitis B and hepatitis C tests during their follow-up.

## Introduction

Hepatitis B virus (HBV) and/or Hepatitis C virus (HCV) co-infection is common among people living with Human Immunodeficiency Virus (HIV) infection (PLWH) because of common routes of transmission such as the exchange of blood or other body fluids during intravenous drug use (IVDU), sexual contact, or mother-to-child transmission during the perinatal period [1, 2]. Such co-infection speeds up the liver disease and raises the morbidity and mortality in PLWH, particularly if left undiagnosed and untreated [3].

HIV continues to be a major public health threat with an additional risk of HBV and/or HCV co-infection. Based on the report of Joint United Nations Programme on HIV/ AIDS 2019 and World Health Organization (WHO) 2017, approximately 38 million people have been diagnosed with HIV, 257 million with HBV, and 71 million with HCV globally at the end of 2020 [4, 5]. The advances in HBV, HCV and HIV therapeutics over the past decades have brought the elimination of their epidemics worldwide to a vast extent. The WHO and the Joint United Nations Programme on HIV/AIDS have implemented strategies to promote the global elimination of these viruses by 2030 [6, 7]. Among other factors, the success of these strategies relies upon the testing and diagnosis of at least 90% of all persons living with HBV, HCV, and HIV infections, as a necessary first step towards engagement in care and treatment [8, 9].

The people living in resource-limited countries with reduced access to diagnosis and medical care are at higher risk of being infected with both viruses [10]. In Nepal, as per the National Immunization Program (NIP), to lessen the burden of HBV, Hepatitis B vaccine is given routinely to every newborn in the form of pentavalent vaccine (DPT-HBsAg-HiB) at 6, 10, and 14 weeks of age [11]. Based on “National HIV Testing and Treatment Guidelines 2020”, all HIV patients should be screened for HBsAg to detect HIV-HBV co-infection, and if negative, should be vaccinated against hepatitis B. The HIV-HBV co-infection should be treated with Tenofovir (TDF) and Lamivudine (3TC) along with antiretroviral therapy. Similarly, all HIV patients should be screened for hepatitis C and should be treated with combinations of sofosbuvir and velpatasvir along with antiretroviral therapy. The preferred first-line regimen for all adults/adolescents, pregnant or breast-feeding women is a three-drug regimen including tenofovir, lamivudine and dolutegravir [12].

The literature regarding the burden of HIV co-infection with HBV and/or HCV in Nepal is sparse. This study aims to analyze prevalence and risk factors associated with co-infection of hepatitis B virus (HBV) and/or hepatitis C virus (HCV) in HIV-positive patient besides their CD4+ T cells status. This study will be helpful in predicting the risk of co-infection in HIV patients and their proper management.

## Materials and methods

### Design

This was a single hospital-based cross-sectional study carried among people living with HIV and attending the hospital for CD4+ T cells count testing. ELISA-based tests that detects HBsAg (J Mitra & Co), and anti-HCV antibody (J Mitra & Co) were carried out after appropriate counselling.

### Setting

B.P. Koirala Institute of Health Sciences (BPKIHS) is a Nepalese autonomous health sciences university. It is located in the Dharan Sub-metropolitan city of Sunsari District. The Institute serves the health education needs of the eastern region of Nepal at the tertiary level.

### Study population

All people living with HIV (PLWH) attending the hospital for CD4+ T cells count testing, and consenting to take part in this study, were included. Those participants who had hepatitis B and/or hepatitis C before the diagnosis of HIV were excluded.

### Sample size and sampling technique

According to a study by Bhattarai et al. in 2018, the prevalence of HIV-HBV, HIV-HCV and HIV-HBV-HCV was 3.6%, 2.9%, and 0.3% respectively [13]. The sample size was calculated considering a maximum prevalence of 3.6%, 5% margin of error and 95% confidence interval by using one proportion formula (https://select-statistics.co.uk/calculators/sample-size-calculator-population-proportion/).

The detailed elaboration is as follows:

$$n=NX/\left(X+N-1\right)\;\text{and}\;X=Z^{2}pq/l^{2}$$

where

n = sample size and N = population size of HIV patients (29503) in Nepal as of 2020 [12].

Z = 1.96 for 95% C.I,

p = prevalence = 0.036; q (compliment of prevalence) = 1-p = 0.964

l = margin of error i.e. 5% of p

Therefore, n = 54

The total sample size calculated was 54. However, we enrolled 486 PLWH over a study period of 12 months (5^th^ May 2019 to 4^th^ May 2020).

### Variables studied

We considered age, gender, mode of HIV transmission, duration of HIV diagnosis, antiretroviral therapy (ART) status, antiretroviral therapy duration, current CD4+ T cells count results, and reports of their hepatitis B and hepatitis C tests.

### Blood workup

Venous blood was tested for CD4+ T cells count using FACSCalibur (BD, USA) and ELISA-based testing for HBsAg (J Mitra & Co, India), and anti-HCV antibody (J Mitra & Co, India) at the Department of Microbiology and Infectious Diseases, BPKIHS.

### Data collection

Relevant demographic, clinical and laboratory data were collected from each individual via face-to-face interview and from hospital records whenever necessary.

### Data entry and analysis

Data were entered into Microsoft Excel 2019 v16.0 (Microsoft, WA, USA) and appropriate commands were used for data cleaning. Entered data was analyzed using Statistical Packages for Social Sciences version 21 (IBM SPSS Corporation, Armonk, New York, USA). Demographic and clinical characteristics of HIV patients were presented as proportions and percentages. The Chi-square test was used to test for group differences. For univariable logistic regression analyses, odds ratios (OR) and 95% Confidence Interval (CI) were calculated. Multivariable logistic regression was used to determine independent risk factors associated with co-infections, and adjusted odds ratios (AOR) were calculated at 95% Confidence Interval (CI). All variables with P < 0.20 were retained in the final multivariable model.

### Ethical approval

Ethical approval was taken from the Institutional Review Board (IRB) of BP Koirala Institute of Health Sciences (Reference No. 577/077/078). Written informed consent was taken from participants before the face-to-face interview. All the participants were explained about the objectives of this research, and the possible risks and benefits of participating in the study. They were also assured that their participation was entirely voluntary, and they could quit the interview at any stage.

## Results

### Demographic and clinical characteristics

We enrolled 486 PLWH over the study period, but 12 PLWH were later excluded as they refused written consent. Out of 474 PLWH, percentage of patients co-infected with HBV, HCV and both HBV-HCV were 2.95% (14/474), 18.14% (86/474) and 2.53% (12/474) respectively.

All HIV patients co-infected with HBV (14, 100.00%), and around half of HIV patients co-infected with HCV (42, 48.84%) belonged to 20–39 years of age group. Half of HIV patients co-infected with both HBV and HCV (6, 50.00%) belonged to 40–59 age group. However, the mean age was comparable across all groups. More than half of HIV patients co-infected with HBV (8, 57.14%) were females. Meanwhile, more than half of HIV patients co-infected with HCV, and both HBV and HCV were males (50, 58.14% and 10, 83.33%).

The primary route of HIV infection was intra-venous drug use (IVDU) in those co-infected with HBV only (8, 57.14%), HCV only (46, 53.49%) and both HBV and HCV (8, 66.67%). More than half of HIV patients co-infected with either HBV or HCV were diagnosed with HIV in the preceding 6–12 months duration (10, 71.44% and 68, 79.07% respectively). Half of HIV patients co-infected with both HBV and HCV (6, 50.00%) were diagnosed with HIV within 6 months. Over four-fifths of all those co-infected with either HBV, HCV, and both HBV and HCV were on ART. Likewise, more than two-thirds of HIV patients co-infected with HBV only, and HCV only (10, 71.44% and 74, 86.05% respectively) were on ART for 6–12 months. Half of HIV patients co-infected with both HBV and HCV (6, 50.00%) were on ART for less than six months. Over four-fifths of HIV patients co-infected with both HBV and HCV (10, 83.33) had CD4 cell count fewer than 200 cells/mm^3^ (Table 1).

**Table 1:** Demographic and clinical characteristics of HIV patients with mono-infection and HBV and/or HCV co-infections (N = 474).

Characteristics	HIV mono-infected	Co-infected with HBV only	Co-infected with HCV only	Co-infected with both HBV & HCV	Total
	N (%)	N (%)	N (%)	N (%)	N (%)
**All**	362(100.00)	14 (100.00)	86 (100.00)	12 (100.00)	474 (100.00)
**Age group (years)**					
**Mean ±SD**	38.43**±**12.08	31.57**±**6.05	36.70**±**9.85	36.67**±**8.25	37.86**±**11.53
0–19	26 (7.18)	0 (00.00)	0 (6.98)	0 (00.00)	26 (5.49)
20–39	146(40.33)	14 (100.00)	46 (53.49)	6 (50.00)	212 (44.72)
40–59	176(48.62)	0 (0.00)	38 (44.19)	6 (50.00)	220 (46.41)
≥60	14 (3.87)	0 (0.00)	2 (2.32)	0 (0.00)	16 (3.38)
**Gender**					
Female	168 (46.41)	8 (57.14)	36 (41.86)	2 (16.67)	214 (45.15)
Male	194 (53.59)	6 (42.86)	50 (58.14)	10 (83.33)	260 (54.85)
**Mode of HIV Transmission**					
IVDU	88 (24.31)	8 (57.14)	46 (53.49)	8 (66.67)	150 (31.65)
Sexual	252(69.61)	6 (42.86)	40 (46.51)	4 (33.33)	302 (63.71)
Vertical	22 (6.08)	0 (00.00)	0(00.00)	0 (00.00)	22 (4.64)
**Duration of HIV diagnosis (months)**					
<6	292 (80.67)	2 (14.28)	4 (4.65)	6 (50.00)	304 (64.14)
6–12	40 (11.05)	10 (71.44)	68 (79.07)	4 (33.33)	122 (25.74)
≥12	30 (8.29)	2 (14.28)	14 (16.28)	2 (16.67)	48 (10.13)
**Ever on ART**					
No	32 (8.84)	2 (14.29)	0 (0.00)	0 (0.00)	34 (7.17)
Yes	330 (91.16)	12 (85.71)	86 (100.00)	12 (100.0)	440 (92.83)
**Duration on ART**					
<6	292 (80.66)	2 (14.28)	4 (4.65)	6 (50.00)	304 (64.14)
6–12	44 (12.15)	10 (71.44)	74 (86.05)	4 (33.33)	132 (27.85)
≥12	26 (7.18)	2 (14.28)	8 (9.30)	2 (16.67)	38 (8.01)
**CD4 (cells/mm** ^**3**^ **)**					
>500	196 (54.14)	0 (0.00)	40 (46.51)	0 (0.00)	236 (49.8)
350–499	76 (20.99)	10 (71.43)	28 (32.56)	2 (16.67)	116 (24.5)
200–349	60 (16.57)	0 (0.00)	12 (13.95)	0 (0.00)	72 (15.2)
<200	30 (8.29)	4 (28.57)	6 (6.98)	10 (83.33)	50 (10.5)

### Association of HIV with HBV, HCV, and both HBV and HCV co-infections

#### HIV-HBV co-infection

In the multivariable model, all PLWH with HIV diagnosis at age equal to and more than 35 years were 80% less likely to get co-infected with HBV than those with age less than 35 years (OR 0.20, 95% CI: 0.06,0.67) (Table 2).

**Table 2:** Proportion and odds ratios of HIV-HBV co-infection in HIV patients (N = 474).

Characteristics	HIV-HBV Co-infection		Univariable Model			Multivariable model##		
	No	Yes	OR	95% CI	P value	AOR	95% CI	P value
**Age group (years)**					**<0.01**			
<35	153(33.26)	10(71.43)	1 (Ref)			1 (Ref)		
≥35	307(66.74)	4(28.57)	0.20	0.06,0.65	<0.01	0.20	0.06,0.67	**<0.01**
**Gender**					0.42			
Female	206(44.78)	8(57.14)	1 (Ref)					
Male	254(55.22)	6(42.86)	0.61	0.21,1.78	0.36			
**Mode of HIV Transmission** ^**1**^					**0.10**			
Sexual	296(64.35)	6(42.86)	1 (Ref)			1 (Ref)		
Others/IVDU	164(35.65)	8(57.14)	2.41	0.82,7.05	0.11	2.13	0.70,6.47	0.18
**Duration of HIV diagnosis (months)**					0.31			
≤12	424(92.17)	12(85.71)	1 (Ref)					
>12	36(7.83)	2(14.29)	1.96	0.42, 9.11	0.39			
**Ever on ART** ^**2**^					0.27			
No	32(6.96)	2(14.29)	1 (Ref)					
Yes	428(93.04)	12(85.71)	0.45	0.10,2.09	0.31			
**Duration of ART**					**0.20**			
≤12	434(94.35)	12(85.71)	1 (Ref)			1 (Ref)		
>12	26(5.65)	2(14.29)	2.78	0.59,13.09	0.20	4.10	0.81,20.81	0.09
**CD4 (cells/mm** ^**3**^ **)**					0.76			
≥350	342(74.35)	10(71.43)	1 (Ref)					
<350	118(25.65)	4(28.57)	1.16	0.36,3.77	0.81			

##Adjusted for age at HIV diagnosis, mode of HIV transmission and duration of ART.

^1^Other mode of transmission is primarily IVDU as there were no cases of HIV-HBV co-infection in PLWH acquired through vertical mode of transmission (Table 1).

^2^Currently on Anti retro-viral therapy (ART).

#### HIV-HCV co-infection

In the multivariable model, HIV patients infected via IVDU were 2.40 times more likely to have HIV-HCV co-infections as compared to those infected via sexual route (OR 2.40, 95% CI: 1.49,3.86) (Table 3).

**Table 3:** Proportion and odds ratios of HIV-HCV co-infection in HIV patients (N = 474).

Characteristics	HIV-HCV Co-infection		Univariable Model			Multivariable model##		
	No (%)	Yes (%)	OR	95% CI	P value	AOR	95% CI	P value
**Age group (years)**					0.27			
<35	129(33.25)	34(39.53)	1 (Ref)					
≥35	259(66.75)	52(60.47)	0.76	0.47,1.23	0.27			
**Gender**					0.50			
Female	178(45.88)	36(41.86)	1 (Ref)					
Male	210(54.12)	50(58.14)	1.18	0.73,1.89	0.50			
**Mode of HIV Transmission** ^**1**^					**<0.01**			
Sexual	262(67.53)	40(46.51	1 (Ref)			1 (Ref)		
Others/IVDU	126(32.47)	46(53.49)	2.39	1.49,3.84	<0.01	2.40	1.49,3.86	**<0.01**
**Duration of HIV diagnosis (months)**					**0.17**			
≤12	360(92.78)	76(88.37)	1 (Ref)			1(Ref)		
>12	28(7.22)	10(11.63)	1.69	0.79, 3.63	0.18	1.71	0.79,3.72	0.18
**Ever on ART** ^**2**^					**<0.01**			
No	34(8.76)	0(0.00)	-			-		
Yes	354(91.23)	86(100.00)	-	-	-	-	-	-
**Duration of ART**					0.59			
≤12	364(93.81)	82(95.35)	1 (Ref)					
>12	24(6.19)	4(4.65)	0.74	0.25,2.19	0.59			
**CD4 (cells/mm** ^**3**^ **)**					0.26			
≥350	284(73.20)	68(79.07)	1 (Ref)					
<350	104(26.80)	18(20.93)	0.72	0.41,1.27	0.26			

##Adjusted for mode of HIV transmission and duration of HIV diagnosis.

^1^Other mode of transmission is primarily IVDU as there were no cases of HIV-HCV co-infection in PLWH acquired through vertical mode of transmission (Table 1).

^2^Currently on Anti retro-viral therapy (ART).

#### HIV-HBV-HCV co-infection

In the multivariable model, HIV patients infected through IVDU were 3.91 times more likely to have HIV-HBV-HCV co-infection than those infected through sexual mode (OR 3.91, 95% CI: 1.05,14.63). HIV patients with CD4 count less than 350 cells/mm^3^ are 13.84 times more likely to have HIV-HBV-HCV co-infection as compared to those with CD4 count ≥350 cells/mm^3^ (OR 13.84, 95% CI: 2.90,66.10) (Table 4).

**Table 4:** Proportion and odds ratios of HIV-HBV-HCV co-infections in HIV patients (N = 474).

Characteristics	HIV-HBV-HCV Co-infection		Univariable Model			Multivariable model##		
	No (%)	Yes (%)	OR	95% CI	P value	AOR	95% CI	P value
**Age group (years)**					0.76			
<35	160(34.63)	3(25.00)	1 (Ref)					
≥35	302(65.37)	9 (75.00)	1.59	0.42,5.95	0.49			
**Gender**					**0.05**			
Female	212(45.89)	2(16.67)	1 (Ref)			1 (Ref)		
Male	250(54.11)	10(83.33)	4.24	0.92,19.57	0.06	2.07	0.41,10.54	0.38
**Mode of HIV Transmission**					**0.03**			
Sexual	298(64.50)	4(33.33)	1 (Ref)			1(Ref)		
Others/IVDU	164(35.50)	8(67.67)	3.63	1.08,12.25	0.04	3.91	1.05,14.63	
**Duration of HIV diagnosis (months)** ^**1**^					0.25			
≤12	426(92.21)	10(83.33)	1 (Ref)					
>12	36(7.79)	2(16.67)	2.37	0.50, 11.22	0.28			
**Ever on ART** ^**2**^					1.00			
No	34(7.36)	0(0.0)	-					
Yes	428(92.64)	12(100.0)	-	-	-			
**Duration of ART**					**0.15**			
≤12	436(94.37)	10(83.33)	1 (Ref)			1 (Ref)		
>12	26(5.63)	2(16.67)	3.35	0.70,16.10	0.13	2.64	0.46,15.16	0.28
**CD4 (cells/mm** ^**3**^ **)**					**<0.01**			
≥350	350(75.76)	2(16.67)	1 (Ref)			1 (Ref)		
<350	112(24.24)	10(83.33)	15.63	3.37,72.38	<0.01	13.84	**2.90,66.10**	**<0.01**

##Adjusted for gender, mode of HIV transmission, duration of ART and CD4^+^ T cells.

^1^Other mode of transmission is primarily IVDU as there were no cases of HIV-HBV-HCV co-infection in PLHIV acquired through vertical mode of transmission (Table 1).

^2^Currently on antiretroviral therapy (ART).

## Discussion

The above study renders the basic demographic and clinical characteristics of HBV and/or HCV co-infections in HIV patients. In this study, the overall prevalence of HBV, HCV or both, with HIV co-infection, was about one-fifth (112, 23.63%). Among these, the proportions of co-infections for HIV-HBV, HIV-HCV and HIV-HBV-HCV were 2.95%, 18.14% and 2.53%.

The findings of our study were consistent with other studies from Nepal. In the first nationally representative study in 2017 by Lonita et al., the prevalence of HBV-HCV co-infection among PLWH in Nepal was 4.4% and 19% respectively [14]. Similarly, in a systematic review study of Nepal from 1990 to 2020, the pooled prevalence of co-infection with HBV, HCV, and combined HBV and HCV was 4.6%, 19.7% and 1.3%, respectively [15]. However, the epidemiological data on HIV-HBV, HIV-HCV and HIV-HBV-HCV co-infections among HIV patients are sparse and variable even within the nation and abroad. The prevalence of HIV-HBV, HIV-HCV, and HIV-HBV-HCV co-infections was 3.62%, 2.93%, and 0.34%, respectively, in a study by Bhattarai et al. [13]. In North India, the prevalence of HBV and HCV co-infection among PLWH was 5.32% and 2.43%, respectively [16]. Similarly, in Hunan Province, China, the prevalence of HIV-HBV, HIV-HCV and HIV-HBV-HCV co-infections among HIV patients was 9.27%, 9.98% and 2.72%, respectively [17]. In Ghana, the prevalence of HIV-HBV, HIV-HCV and HIV-HBV-HCV co-infections among HIV patients was 12.5%, 5.5% and 18.0% respectively in a study by Boateng et al., and was 6.1%, 0.5% and 0.0% respectively in a study by Pappoe et al. [18, 19]. Based on these studies; the prevalence of HIV-HCV was very high in our study but consistent with multiple other studies. One possible reason could be that the IVDU route of HIV transmission could be playing a significant role in the transmission of HCV compared to sexual route, which is also evident from our study. Looking at the burden of HIV-HCV, the National Center for AIDS and STI Control (NCASC) implemented Hepatitis C (HCV) treatment on 21^st^ March among HIV-HCV co-infection from three ART centers of Kathmandu Valley of Nepal [12].

In our study, all PLWH at age equal to and more than 35 years were less likely to be infected with HIV-HBV co-infection than those under 35 years. This finding is in contrast to the study by Choy et al. which showed that age 30–49 and more than 50 years was significantly associated with HIV-HBV co-infection [20]. This lower proportion of HIV-HBV co-infection among elder HIV patients could be because of minor frequency of higher aged (≥35 years) HIV patients in this category. But the exact reason needs to be elucidated from other prospective cohort studies.

Our result shows a high likelihood of males having HIV-HBC-HCV co-infections compared to females. This finding aligns with the first nationally representative study in 2017 by Lonita et al., where the males with HIV were 5.7 times more likely to had HIV-HCV co-infection [11]. This might be because of sexual promiscuity in male and high-risk behavior like IVDU in male. More than three-fourth of HIV patients with HIV-HBV-HCV triple co-infections were male in our study.

In our study, HIV patients who acquired HIV through IVDU route were 2.13 times more likely to have HIV-HBV co-infection as compared to those with sexual mode. Similarly, HIV patients infected via IVDU mode were more than two times more likely to have HIV-HCV and HIV-HBV-HCV co-infections as compared to those with sexual mode. This findings are statistically significant and are congruent with other studies from Nepal and abroad where IVDU were more likely be co-infected with HBV and HCV [11, 21, 22]. The likely explanation could be that intravenous drugs use poses higher risk of transmission of blood-borne infections [23, 24].

To our knowledge, the data regarding association between CD4+ T cells count and HIV-HBV-HCV co-infections in Nepal is scarce. Our study showed that HIV patients with CD4^+^ T cells count less than 350 cells/mm^3^ were at 13.84 times higher risk of having triple co-infections as compared to patients with CD4+ T cells more than 350 cells/mm^3^. This is in accordance with a study by Bhattarai et al. which showed that HIV patients with CD4+ T cells more than 200 200 cells/mm^3^ were 81% less likely to have HIV-HCV co-infection [13]. The depleting CD4^+^ T cells count is a marker of immune dysfunction and HIV progression [25, 26] and indicators of acquiring multiple opportunistic infections and co-infections.

Our study has certain limitations. We did not assess the prevalence of other hepatitis viruses in the HIV positive population, such as hepatitis A, hepatitis D, and hepatitis E. We did not differentiate the sexual mode of transmission into heterosexual or homosexual practices. We did not categorize the co-infections for HIV-HBV, HIV-HCV and HIV-HBV-HCV patients based on marital status, ethnicity and educational qualifications.

## Conclusion

The HBV and/or HCV co-infection among HIV patients is a significant health threat in Nepal. The physicians involved in care of HIV/AIDS should be vigilant to screen for these infections frequently. The policy makers should integrate the Hepatitis B vaccination into the HIV prevention program. Similarly, the rapid initiation of ART in those diagnosed with HIV can help to maintain CD4+ T cells count at the optimum level, and this can help to bring down the HIV-HBV-HCV co-infection in them. Besides, patients with HIV need proper counseling and awareness about their disease so that the patients understand the risks of blood-borne disease and transmission due to unhygienic needles in IV drug use and engagement in unsafe sexual intercourse.

## Supporting information

S1 Data (XLSX)

## Abbreviations

ART: Antiretroviral Therapy
CD: Cluster of Differentiation
HBV: Hepatitis B Virus
HCV: Hepatitis C Virus
HIV: Human Immunodeficiency Virus
IVDU: Intra-Venous Drug Use
PLWH: People Living With HIV
STI: Sexually Transmitted Infections
WHO: World Health Organization
